# The structural and optical constants of Ag_2_S semiconductor nanostructure in the Far-Infrared

**DOI:** 10.1186/s13065-015-0099-y

**Published:** 2015-05-22

**Authors:** Reza Zamiri, Hossein Abbastabar Ahangar, Azmi Zakaria, Golnoosh Zamiri, Mehdi Shabani, Budhendra Singh, J M F Ferreira

**Affiliations:** Department of Physics, Faculty of Science, Universiti Putra Malaysia, Serdang, Selangor 43400 UPM Malaysia; Department of Materials and Ceramic Engineering (DEMaC), University of Aveiro, Campus Santiago, Aveiro, 3810-193 Portugal; Department of Chemistry, Faculty of Science, Najafabad Branch, Islamic Azad University, Najafabad, Isfahan Iran; TEMA-NRD, Mechanical Engineering Department and Aveiro Institute of Nanotechnology (AIN), University of Aveiro, Aveiro, 3810-193 Portugal

**Keywords:** Nanostructures, Semiconductors, Raman spectroscopy, Infrared spectroscopy, Crystal structure, Optical properties

## Abstract

**Background:**

In this paper a template-free precipitation method was used as an easy and low cost way to synthesize Ag_2_S semiconductor nanoparticles. The Kramers–Kronig method (K–K) and classical dispersion theory was applied to calculate the optical constants of the prepared samples, such as the reflective index *n(ω)* and dielectric constant *ε(ω)* in Far-infrared regime.

**Results:**

Nanocrystalline Ag_2_S was synthesized by a wet chemical precipitation method. Ag_2_S nanoparticle was characterized by X-ray diffraction, Scanning Electron Microscopy, UV-visible, and FT-IR spectrometry. The refinement of the monoclinic β-Ag2S phase yielded a structure solution similar to the structure reported by Sadanaga and Sueno. The band gap of Ag_2_S nanoparticles is around 0.96 eV, which is in good agreement with previous reports for the band gap energy of Ag_2_S nanoparticles (0.9–1.1 eV).

**Conclusion:**

The crystallite size of the synthesized particles was obtained by Hall-Williamson plot for the synthesized Ag_2_S nanoparticles and it was found to be 217 nm. The Far-infrared optical constants of the prepared Ag_2_S semiconductor nanoparticles were evaluated by means of FTIR transmittance spectra data and K–K method.

**Graphical abstract:**
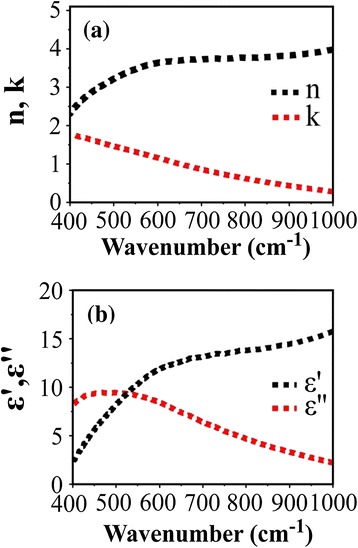
The Far-infrared optical constants of Ag2S semiconductor nanoparticles.

## Background

In recent years, nanometer-sized chalcogenide semiconductors have drawn attention as a component of nanotechnology, mainly due to their physical and chemical properties, heavily dependent on their shape and size. The Ag_2_S is found amongst the most important chalcogenides and because of its unique optoelectronic properties. It have been extensively studied due to its many potential applications in optical and electronic devices such as infrared detectors, photoconductive cells, magnetic field sensors and photoconductors, amongst others [1–5]. Ag_2_S is an effective semiconductor material due to a large absorption coefficient and a direct band gap of 0.9 to 1.05 eV. It is a coinage mineral undergoes a structural phase transition. Above 183 °C, Ag_2_S appear with a cubic structure known as argentite (α-Ag_2_S). At room temperature, Ag_2_S have a monoclinic structure named acanthite, space group P2_1_/c and Z = 4 (β-Ag_2_S) [6, 7]. The α-Ag_2_S behaves like a metal (dσ/dT < 0) while β-Ag_2_S behaves like a semiconductor (dσ/dT > 0, with activation energy of 1.3 eV) [8–10]. Several methods have been developed for the synthesis of Ag_2_S nanoparticles such as solvothermal method, hydrothermal route, and single-source precursor routes [11]. Yu *et al.* synthesized sub-micrometer Ag_2_S particles thru a simple hydrothermal method but it is difficult to control the size and shape of the nanoparticles for the large-scale synthesis of high-quality nanoparticles [12]. Qin *et al.* successfully synthesized Ag_2_S nanorods by a biomimetic route in the lysozyme solution at physiological temperature and atmospheric pressure [4]. In another work, Wang *et al.* synthesized spherical silver sulphide nanoparticles (Ag_2_S) at 205 °C under N_2_ atmosphere by a direct reacting silver acetate with n-dodecanethiol [13]. Therefore, there is a considerable challenge for the synthesis of Ag_2_S nanoparticles on a large scale through a simple and low-cost approach.

In this paper, a template-free precipitation method was used to prepare nanometric powders of Ag_2_S. The structural and optical constants of the prepared Ag_2_S nanometric powders in Far infrared were calculated and are presented for the first time.

### Experimental section

#### Synthesis and characterization of Ag_2_S nanoparticles

**Fig. 1:**
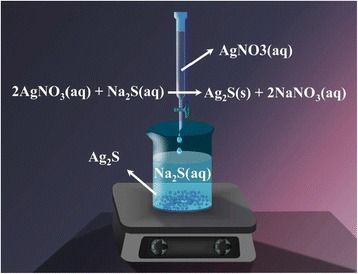
Schematic diagram of experimental set up

Nanocrystalline Ag_2_S was synthesized by a wet chemical precipitation method. Initially 0.1 mmol of AgNO_3_ (Aldrich, Germany) was dissolved in 50 ml of distilled water. The obtained solution was added drop wise into 50 mL 0.1 M Na_2_S solution. Finally, the as prepared precipitated nanocrystalline powder was collected and dried after centrifugation at 80 °C during a 3 h period. The schematic diagram for the experimental set up and chemical reaction is shown in Fig. 1. The structure and morphology of the sample was studied by X-ray diffraction (Shimadzu XRD-6000, Tokyo, Japan) and Scanning Electron Microscopy (SEM, SU-70, Hitachi). The study of the optical properties of the samples was carried out by UV–visible (Perkin-Elmer, Lambda 35) and FT-IR spectrometry.

## Results and discussion

### Phase and compositional study (XRD)

**Fig. 2:**
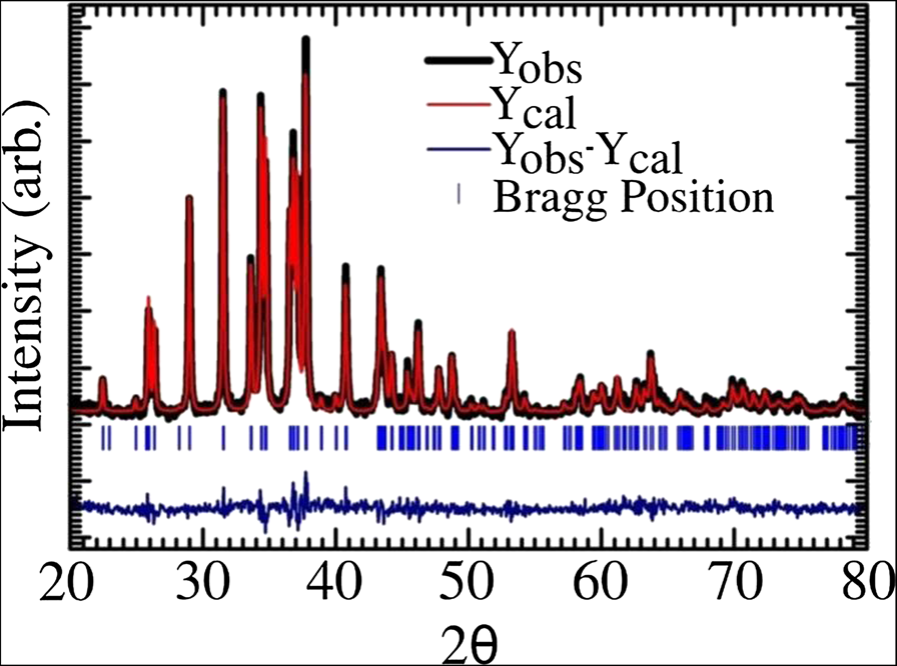
X-Ray Diffraction patterns and Rietveld refinement plot for Ag_2_S nanoparticles powder

Figure 2 shows the X-ray diffraction pattern for synthesised Ag_2_S particles. A Rietveld refinement analysis was performed after x-ray diffraction pattern acquisition. The refinement of the monoclinic β-Ag2S phase yielded a structure solution similar to the structure reported by Sadanaga and Sueno [8]. No impurity phase was observed in the X-ray diffraction pattern. However, the refined structure from this study showed a slight deviation in the xyz coordinates for Ag and S atom. The refined parameters are listed in Table 1, and the Rietveld refinement diffraction pattern of β-Ag2S structure is shown in Fig. 2.

**Table 1:** Structural details and refined parameters obtained by Rietveld refinement

Basic structural details						
Structure				Space group		
*Monoclinic*				*P 21/c*		
Lattice parameters (in Å) and angle (in °)						
*a*	*b*	*c*	*α*	*β*	*γ*	*Vol. (Å3)*
4.2278	6.9289	9.5323	90	125.58	90	227.11
Atomic coordinates parameters						
*Atom*	*x/a*		*y/b*	*z/c*		*SOF*
Ag1	0.07245		0.01478	0.30895		1
Ag2	0.72498		0.32529	0.43819		1
S1	0.49293		0.23577	0.13261		1
Anisotropic displacement parameters, in Å2						
*Atom*	*U*_*11*_	*U*_*22*_	*U*_*33*_	*U*_*12*_	*U*_*13*_	*U*_*23*_
Ag1	0.03732	0.04222	0.05706	0.01489	0.03081	0.01655
Ag2	0.05167	0.05616	0.03745	-0.01454	0.04492	-0.00585
S1	0.01985	0.01236	0.00734	0.01186	0.02756	-0.00555
Other parameters						
*R*_*p*_	*R*_*wp*_	*R*_*exp*_	*R*_*b*_	*R*_*f*_	*χ* ^*2*^	*c/a*
18.8	22.7	20.14	7.94	6.72	1.27	2.2547
Goodness of fit						
D-W statistics (d)			Q_D_ = expected (d)	S (goodness of fit) = R_wp_/R_exp_		
1.6094			1.8251	1.13		

To determine the strain and size effect associated to the synthesized Ag_2_S particles, Hall-Williamson method was used as the estimation of the particle size. This is explained by the Scherrer equation not taking in consideration for the broadening due to lattice strain presence. Generally, the observed peak broadening B_o_ can be attributed to

1 $$ {\mathrm{B}}_{\mathrm{r}} = {\mathrm{B}}_{\mathrm{o}} - {\mathrm{B}}_{\mathrm{i}} $$

where B_o_ is the observed peak broadening in radians, B_i_ is the instrumental broadening in radians, and B_r_ is the broadening due to the small particle size and lattice strain. Using the Scherrer equation, the broadening caused by small crystallite size may be expressed as:

2 $$ {B}_C = \frac{k\lambda }{d\  cos\theta} $$

where: B is the broadening solely caused by small crystallite size, k is a constant whose value depends on particle shape and is usually taken as unity, d is the crystallite size, θ is the Bragg angle and λ is the wavelength of the incident X-ray beam (1.5418° A). Similarly, according to Wilson, the broadening caused by lattice strain is expressed as:

3 $$ {B}_s=4\varepsilon \tan \theta $$

where: B is the peak broadening caused by the lattice strain, ε the strain distribution within the material and θ is the Bragg angle [14]. The instrumental broadening was estimated performing a XRD to a pure strain-free silicon standard under identical conditions. The total broadening excluding the instrumental broadening of the peak is expressed as the sum of eqn (2) and (3) [15]:

4 $$ {B}_r=\frac{k\lambda }{t\  Cos\theta} + 4\ \varepsilon\ tan\theta $$

5 $$ \frac{Br\  Cos\theta}{\lambda }=\frac{k}{t} + \varepsilon\ \frac{4 Sin\theta}{\lambda } $$

**Fig. 3:**
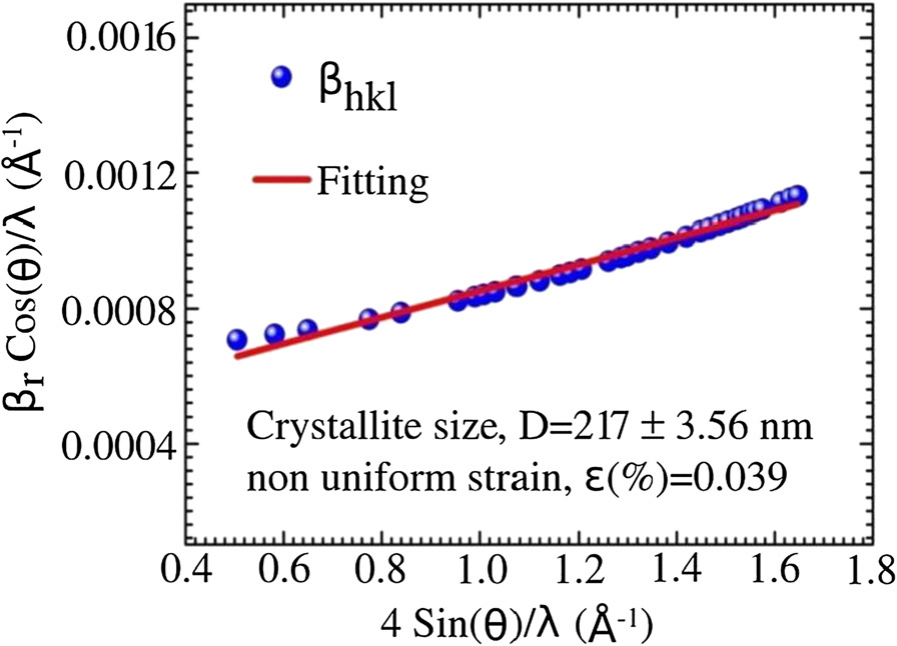
W-H analysis of Ag_2_S particles

The plot of B_r_ cos(θ)/λ versus 4sin(θ)/λ is a straight line with slope equal to ε and hence the particle size can be estimated from the intercept. A typical Hall-Williamson plot for the synthesized Ag_2_S nanoparticles is shown in Fig. 3.

The crystallite size of the synthesized particle was found to be 217 nm. A small non uniform lattice strain (0.039 %) was observed in the sample. The non-uniform strain and the crystallite size was calculated from the slope and the y-intercept of the fit, respectively.

### Morphology study (SEM)

**Fig. 4:**
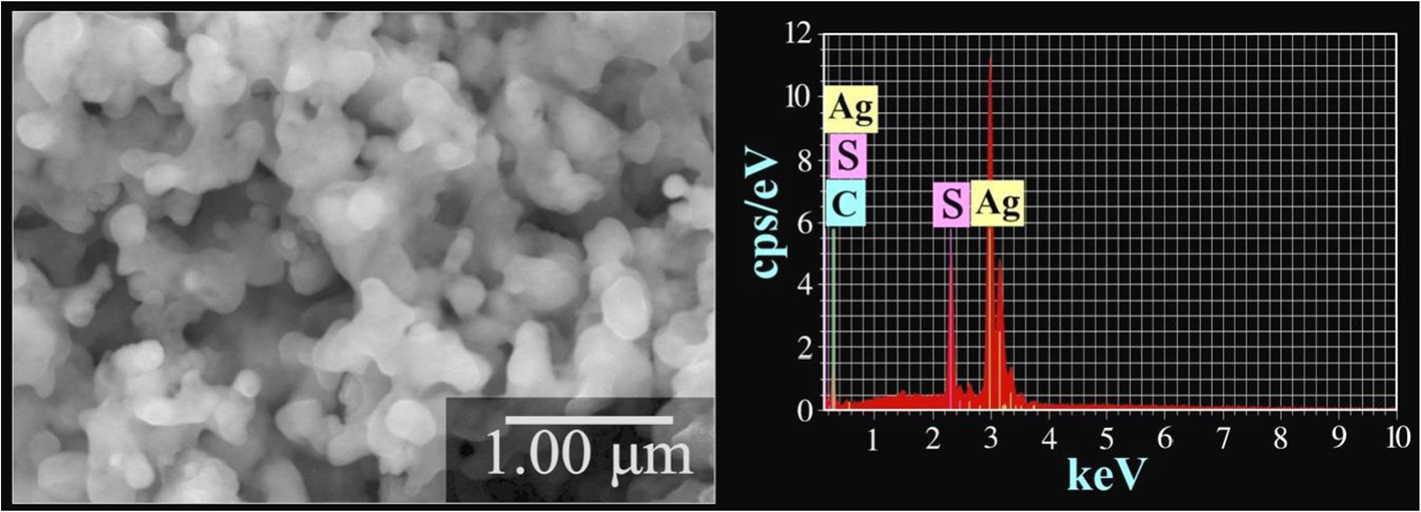
SEM image (left side) of and EDS analysis of the Ag_2_S nanoparticle (right side)

Figure 4 (left) depicts the SEM image of Ag_2_S nanoparticles. Formation of agglomerated spherical Ag_2_S nanoparticles can be seen from this Figure. Therefore it is difficult to estimate the real particles size. Energy dispersive X-ray spectroscopy (EDS) was also performed to determine the chemical composition of the prepared Ag_2_S nanoparticles (shown in Fig. 4 (right)). The obtained EDS results confirmed the presence of Ag and S in the final products.

The absence of extra peaks, besides the expected ones for nanocrystals, suggests that the obtained powders are very pure.

### UV–VIS reflectance

**Fig. 5:**
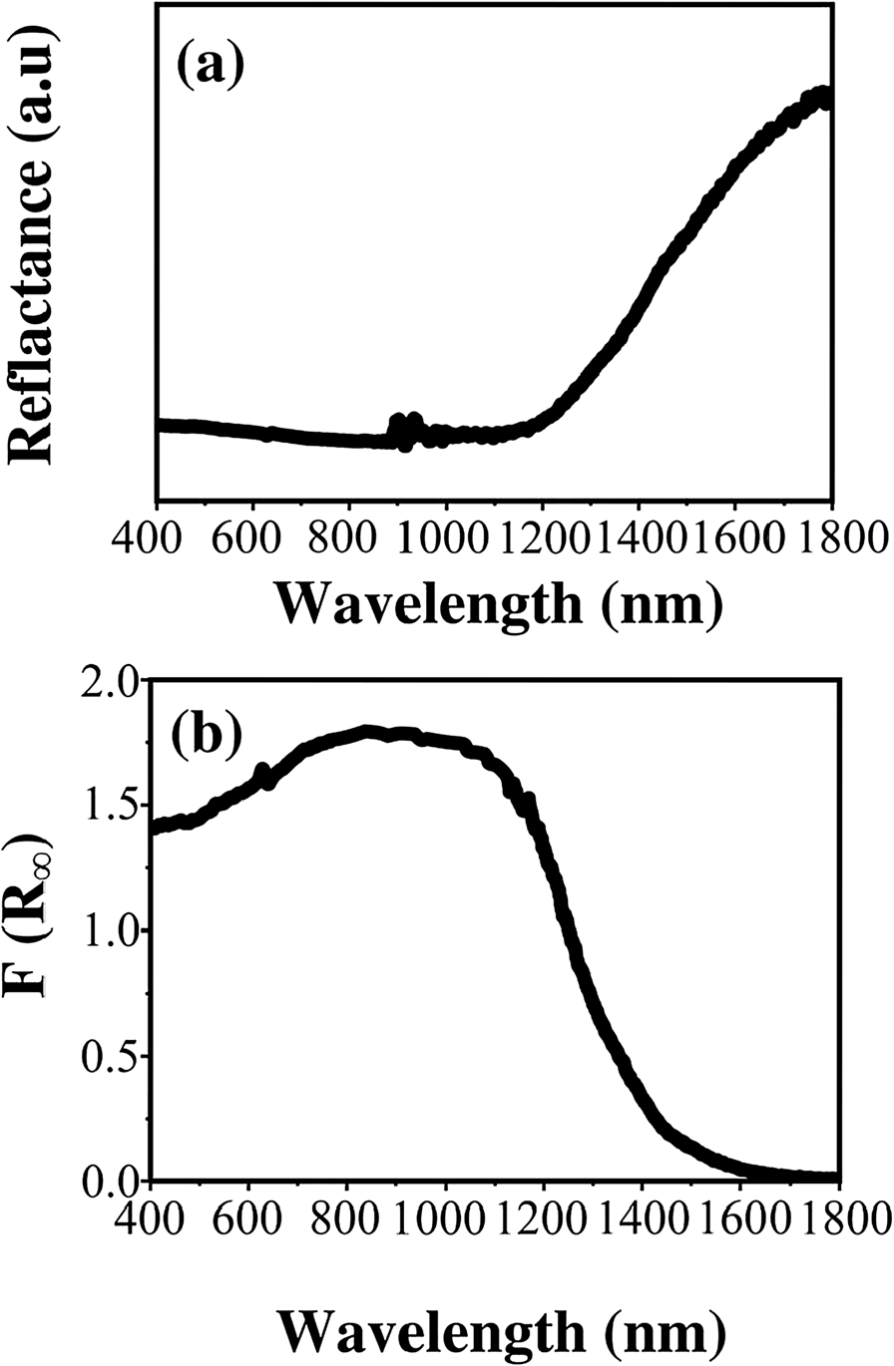
UV–VIS reflectance spectrum of Ag2S nanoparticles **a** reflectance and **b** absorption coefficient

The UV–VIS reflectance spectrum of the sample is presented in Fig. 5a. The Kubelka–Munk function was used to convert the diffuse reflectance into the absorption coefficient and spectrum is presented in Fig. 5b.

6 $$ \alpha =\frac{k}{s}=\frac{{\left(1-{R}_{\infty}\right)}^2}{2{R}_{\infty }}\equiv F\left({R}_{\infty}\right) $$

where S and K are the scattering and absorption coefficients; the reflectance *R*_*∞*_ is equal to: $\frac{R_{sample}}{R_{S \tan dard}}$ [16].

Bulk Ag_2_S is a semiconductor with a direct band gap of 0.9 to 1.05 eV [17]. The following equation was used to determine the band gap of Ag_2_S nanoparticles [18]:

7 $$ \alpha =A{\left(h\nu -{E}_g\right)}^n/h\upsilon $$

where A is constant, E_g_ is the absorption band gap, *α* is the absorption coefficient, and *n* depends on the type of transition, *n* may assume the values 1/2, 2, 3/2 and 3 respectively corresponding to allowed direct, allowed indirect, forbidden direct and forbidden in direct transitions [19].

**Fig. 6:**
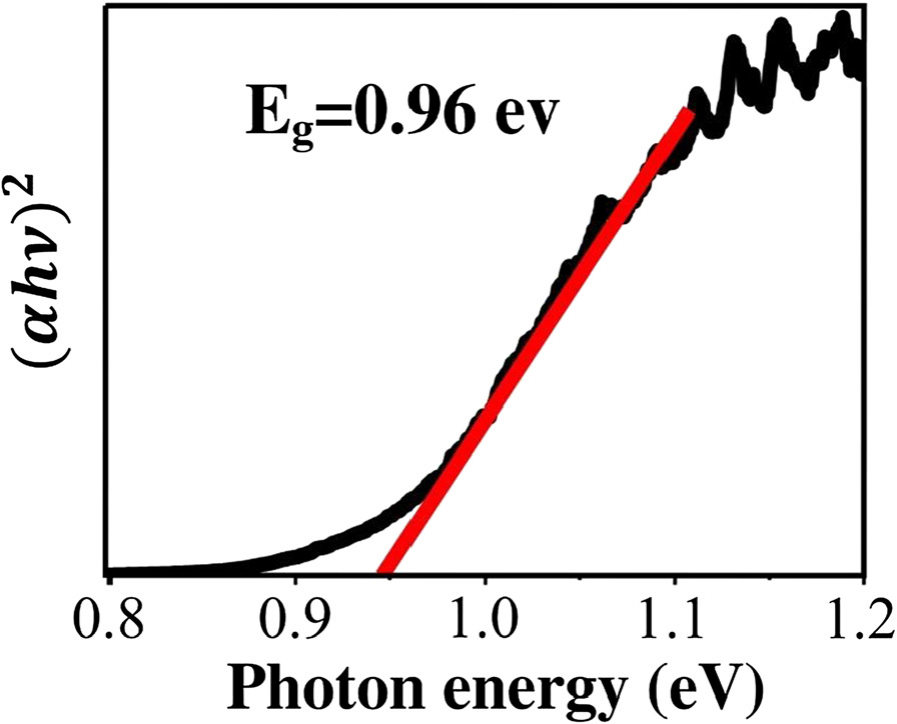
(α*h*ν)^2^ versus *h*ν for Ag_2_S nanoparticles

Since Ag_2_S nanoparticles have direct allowed transitions so we choose *n* = 1/2. The band gap of Ag_2_S nanoparticles was determined by extrapolating the function of (*αhυ*)^2^ in term of *hυ* as shown in the Fig. 6 and it was found that the band gap of Ag_2_S nanoparticles is around 0.96 eV, which is in good agreement with previous reports for the band gap energy of Ag_2_S nanoparticles (0.9–1.1 eV) [19–21].

### FT-IR analysis

**Fig. 7:**
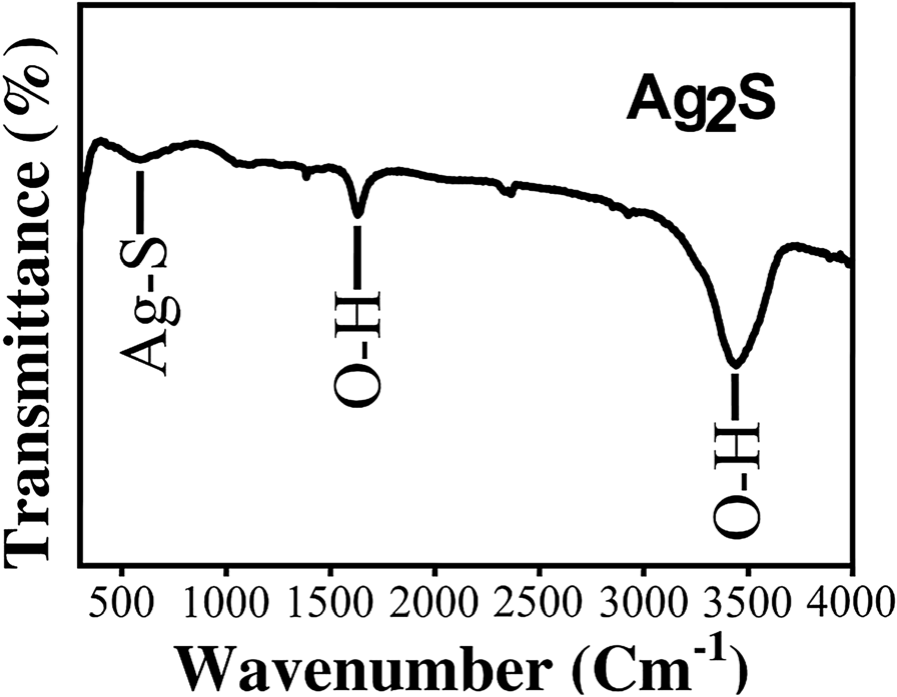
FT-IR spectrum of the Ag_2_S nanoparticles

Figure 7 shows FT-IR spectrum of Ag_2_S nanometric powders. The characteristic vibration of Ag–S appears located at 500–600 cm^−1^ while the broad and small peaks located at 3400 and 1600 cm^−1^ can be attributed to the stretching and bending vibrations of the O–H bond of the adsorbed H_2_O molecules on the surface of Ag_2_S [22, 23].

#### Optical constants of Ag_2_S nanoparticles

The K–K method was used to determine the Far-infrared optical constants of the prepared Ag_2_S semiconductor nanometric powders by using FT-IR transmittance spectral data. The absorption (A) can be obtained from transmittance according to Lambert’s law [24]:

8 $$ A\left(\omega \right)= log\frac{I_0}{I}=lo{g}_{10}\frac{1}{T\left(\omega \right)}=2-lo{g}_{10}\left(T\left(\omega \right)\%\right) $$

9 $$ R\left(\omega \right)=100-\left[T\left(\omega \right)+A\left(\omega \right)\right] $$

where *R(ω)* is the reflectance in the particular wave number. The reflective index *n* is an important physical quantity in optical design and generally is a complex quantity:

10 $$ \tilde{n}\ \left(\omega \right)=n\left(\omega \right)+ik\left(\omega \right) $$

where *n(ω)* and *k(ω)* are the real and the imaginary parts of complex refractive index respectively, and can be obtained by the following equations:

11 $$ n\left(\omega \right)=\frac{1-R\left(\omega \right)}{1+R\left(\omega \right)-2\sqrt{R\left(\omega \right)} \cos \varphi \left(\omega \right)} $$

12 $$ k\left(\omega \right)=\frac{2\sqrt{R\left(\omega \right)} \cos \left(\varphi \right)}{1+R\left(\omega \right)-2\sqrt{R\left(\omega \right)}\mathrm{cosb}\ \varphi \left(\omega \right)} $$

Here, *φ(ω)* is the phase change between the incident and the reflected signal at a particular wavenumber *ω*. This phase change can be calculated from the K–K dispersion relation [25]:

13 $$ \varphi \left(\omega \right)=\frac{-\omega }{\pi }{\displaystyle \underset{0}{\overset{\infty }{\int }}}\frac{LnR\left({\omega}^{\hbox{'}}\right)-LnR\left(\omega \right)}{\omega^{\hbox{'}2}-{\omega}^2}\ d{\omega}^{\prime } $$

This integral can be precisely evaluated by Maclaurin’s method [26]:

14 $$ \varphi \left({\omega}_j\right)=\frac{4{\omega}_j}{\pi}\times \varDelta \omega \times {\displaystyle \sum_i}\frac{ \ln \left(\sqrt{R\left(\omega \right)}\right)}{\omega_i^2-{\omega}_j^2} $$

here *Δω* = *ω*_*j* + 1_ − *ω*_*j*_ and if *j* is an even number then *i*=1, 3, 5, 6,,…*j* − 1, *j* + 1, ….. while if *j*is an odd number then *i* =2, 4, 6,…*j* − 1, *j* + 1, …..

In addition, the dielectric function can be obtained by the square of the refractive index. Therefore, the real and imaginary parts of the complex dielectric function are:

15 $$ \overline{\varepsilon}={\left[\tilde{n}\left(\omega \right)\right]}^2={\left[n\left(\omega \right)+ik\left(\omega \right)\right]}^2 $$

16 $$ \Rightarrow\ {\varepsilon}^{\prime }+i{\varepsilon}^{{\prime\prime} }={n}^2\left(\omega \right)-{k}^2\left(\omega \right)+2 in\left(\omega \right)k\left(\omega \right) $$

17 $$ \Rightarrow\ \left\{\begin{array}{c}\hfill {\varepsilon}^{\prime}\left(\omega \right) = {n}^2\left(\omega \right)-{k}^2\left(\omega \right)\kern6.5em \hfill \\ {}\hfill {\varepsilon}^{{\prime\prime}}\left(\omega \right)=2n\left(\omega \right)k\left(\omega \right)\kern7.75em \hfill \end{array}\right. $$

**Fig. 8:**
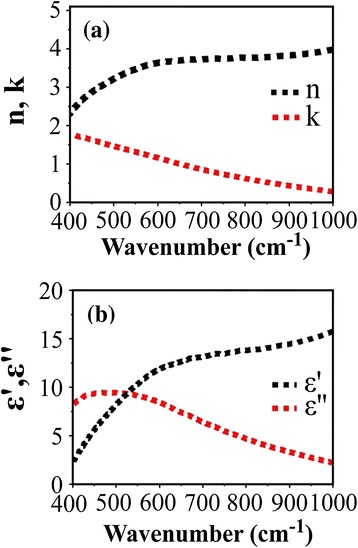
The Far-infrared optical constants of Ag_2_S semiconductor nanoparticles **a** refractive index and extinction coefficient, **b** real and imaginary parts of dielectric functions

The Far-infrared optical constants of Ag_2_S semiconductor nanoparticles was calculated by the above equations and the spectrums are presented in Fig. 8a and 8b.

## Conclusion

We have successfully prepared Ag_2_S semiconductor nanometric powders by using a simple and low cost wet chemical precipitation technique. The micro-structural analysis of the sample was done through XRD pattern analysis and Rietveld refinement analysis. No impurity phase was observed in the X-ray diffraction pattern. The crystallite size of the synthesized particles was obtained by Hall-Williamson plot for the synthesized Ag_2_S nanoparticles and it was found to be 217 nm. The Far-infrared optical constants of the prepared Ag_2_S semiconductor nanoparticles were evaluated by means of FTIR transmittance spectra data and K–K method.
